# Detection of Specific IgA Antibodies against a Novel Deamidated 8-Mer Gliadin Peptide in Blood Plasma Samples from Celiac Patients

**DOI:** 10.1371/journal.pone.0080982

**Published:** 2013-11-22

**Authors:** Sara Vallejo-Diez, David Bernardo, María de Lourdes Moreno, Alba Muñoz-Suano, Luis Fernández-Salazar, Carmen Calvo, Carolina Sousa, José A. Garrote, Ángel Cebolla, Eduardo Arranz

**Affiliations:** 1 Mucosal Immunology Laboratory, IBGM, University of Valladolid-Consejo Superior de Investigaciones Científicas, Valladolid, Spain; 2 Department of Microbiology and Parasitology, University of Seville, Seville, Spain; 3 Biomedal S.L, Seville, Spain; 4 Gastroenterology Service, Hospital Clínico Universitario, Valladolid, Spain; 5 Pediatric Service, Hospital Clínico Universitario, Valladolid, Spain; 6 Clinical Laboratory, Hospital Universitario Rio Hortega, Valladolid, Spain; Tulane University, United States of America

## Abstract

We studied whether celiac disease (CD) patients produce antibodies against a novel gliadin peptide specifically generated in the duodenum of CD patients by a previously described pattern of CD-specific duodenal proteases. Fingerprinting and ion-trap mass spectrometry of CD-specific duodenal gliadin-degrading protease pattern revealed a new 8-mer gliadin-derived peptide. An ELISA against synthetic deamidated 8-mer peptides (DGP 8-mer) was used to study the presence of IgA anti-DGP 8-mer antibodies in plasma samples from 81 children (31 active CD patients (aCD), 17 CD patients on a gluten-free diet (GFD), 10 healthy controls (C) and 23 patients with other gastrointestinal pathology (GP)) and 101 adults (16 aCD, 12 GFD, 27 C and 46 GP-patients). Deamidation of the 8-mer peptide significantly increased the reactivity of the IgA antibodies from CD patients against the peptide. Significant IgA anti-DGP 8-mer antibodies levels were detected in 93.5% of aCD-, 11.8% of GFD- and 4.3% of GP-patients in children. In adults, antibodies were detected in 81.3% of aCD-patients and 8.3% of GFD-patients while were absent in 100% of C- and GP-patients. Duodenal CD-specific gliadin degrading proteases release an 8-mer gliadin peptide that once deamidated is an antigen for specific IgA antibodies in CD patients which may provide a new accurate diagnostic tool in CD.

## Introduction

 Celiac disease (CD) is a gluten-sensitive enteropathy that develops in genetically susceptible individuals following exposure to dietary wheat gluten and similar proteins from barley, rye and some varieties of oats [1–3] (Highlights S1).

 Prolamins constitute eighty percent of total gluten proteins. They are soluble in ethanol and rich in glutamine (Q) and proline (P) residues. Their names varies based on the source cereal (gliadin from wheat, secalin from rye, hordein from barley and avenin from oats) and they are classified in α-, γ- and ω-prolamins according to their electrophoretic mobility. The remaining 20% of the total gluten proteins are insoluble in ethanol and are divided in high molecular weight (HMW) and low molecular weight (LMW) glutenins.

 CD is characterized by villous atrophy, crypt hyperplasia and infiltration of inflammatory cells, both in the epithelium and in the mucosal lamina propria of the small intestine. The disease might affect approximately 1% of the Caucasian population. At present, the only treatment for CD is a life-long strict gluten-free diet (GFD), which in most cases leads to a complete remission of the disease. 

 The inflammatory reaction appears to be driven by activation of Th1-like-CD4+ T cells that recognize gluten peptides modified by the enzyme tissue transglutaminase (tTG) in the context of human histocompatibility leucocyte antigen (HLA) region namely the HLA-DQ2/DQ8 molecules [4,5]. Deamidation is important for binding of gliadin-derived peptides to HLA DQ2/DQ8 molecules and subsequently for the stimulation of T cells [4]. Several gliadin-derived peptides have been identified as ligands for the disease-associated HLA-DQ molecules [6]. Whereas the T cell response in CD is relatively well understood, less is known about the B cell response [7]. Mucosal B cells are triggered to produce antibodies against food antigens, anti-gliadin (AGA), anti-deamidated gliadin peptides (DGP); and against self molecules as tTG. At the mucosal compartments humoral responses are mainly mediated by IgA antibodies so they are more specific than IgG antibodies as serological markers in gastrointestinal diseases like CD. 

 The diagnosis of CD is based on 3 pillars: i) mucosal alterations as determined by histological evaluation of duodenal biopsy, ii) genetic susceptibility (HLA-DQ2/DQ8) and iii) a positive serology (antibodies against tTG and anti-endomisium) [8]. Despite small bowel biopsy is still the gold standard for CD diagnosis, endoscopy is uncomfortable and expensive. Therefore, research has been focused on developing less-invasive markers for its correct diagnosis. 

 Many approaches have led to the identification of several gluten peptides that can stimulate T cells from CD patients. Such peptides were found in α-, γ- and ω-gliadins as well as in glutenins. While α- and ω-gliadin-derived peptides are immunodominant in adults, responses to the LMW glutenins and γ-gliadins are frequently observed in children [9,10]. The study however of gliadin-derived peptides specifically generated in the duodenum of CD patients improves our current knowledge about gluten peptides since they may be used for development of specific serological markers with a clinical application and/or to target them in vivo to prevent their immunogenic properties in the celiac duodenum.

 We have previously described for the first time to our knowledge a specific pattern of gliadin-degrading metalloproteases in the duodenal mucosa of CD patients, not found in the duodenum from non-CD patients [11]. Therefore CD patients (both aCD and GFD-patients) who have gliadin intolerance, carry CD-specific duodenal gliadin-degrading proteases suggesting a potential implication for such enzymes in CD pathogenesis. 

 The origin of the gliadin-degrading activity in CD is still unknown. One of the most accepted hypothesis is the possible contribution of specific CD mucosal-associated bacteria [11] which could be explained by the differences in the microbiota from patients with active celiac disease and on a GFD reported recently [12].

 In order to study the possible implication of these proteases, independently of their origin, in the development of CD, here we studied whether a 8-mer gliadin peptide released by the action of duodenal CD-specific gliadin-degrading proteases could be specific epitopes of IgA antibodies found in the plasma of CD patients. We also studied if deamidation of the 8-mer gliadin peptide increased its reactivity against the IgA Abs and if the latter were altered in CD patients following a GFD. 

 In this manuscript we report for the first time to our knowledge an 8-mer gliadin-derived peptide specifically generated in the CD duodenum, that likely deamidated by tTG, is an epitope of specific IgAs found in plasma samples from CD patients, providing a potential utility as diagnostic biomarkers and/or markers to monitor GFD compliance.

## Material and Methods

### 1: Study patients

 Four groups of patients have been included in this study: active CD-patients (aCD), CD-patients on a gluten-free diet (GFD), healthy controls (C) and patients with other gastrointestinal pathology (GP). The first one (aCD) included celiac patients with a recent diagnosis of the disease, therefore on a normal gluten-containing diet. They had clinical symptoms, positive serology (IgA anti-TGt and/or IgA anti-endomisium (EmA) antibodies), genetic susceptibility (HLA-DQ2/DQ8) and all but one had compatible lesion in the duodenal biopsy (Marsh I-III). Remainder cases fulfilled the ESPGHAN criteria (2012) to avoid intestinal biopsy. Celiac patients on a gluten-free diet (GFD), presented a reduction of symptoms, a normalization of the serological markers and a recovery of the histological lesion. Patients with other gastrointestinal pathology (PGI) were patients with a clinical profile compatible with CD but where diagnosis was later discarded, suffering another intestinal disease. These patients had negative serology and mild mucosal alterations non compatibles with CD. Finally, a group of healthy controls was included. Among each study group, patients were separated according to the age in adults (mean age 35) and children (mean age 7). 

 Tissue and plasma samples included in this retrospective study (2004-2012) were obtained from the Gastroenterology and Pediatric Services from the *Hospital Clínico Universitario* and the *Hospital Universitario Rio Hortega*, Valladolid, Spain. Written consent was obtained from adult patients and parents or legal tutors from children. The study protocol was approved by the Ethics Committee of both, the *Hospital Clínico Universitario* and the Faculty of Medicine, University of Valladolid. 

####  Biopsy samples

tissue samples (Table 1) were obtained according to the revised ESPGHAN criteria [13] from aCD-patients (n=2), GFD-patients (n=2) and GP-patients (n=2). All groups included one child and one adult. Duodenal explants were immediately submerged in 0.5 ml of RNALater® solution (Ambion Inc, Texas, USA) and stored at -20°C until protein extraction. 

**Table 1:** Patients groups from biopsy samples included in this study according to revised ESPGHAN criteria.

	**Study patients**	**n**	**Children**	**Adults**	**HLA DQ2/DQ8**	**IgA-tTG/EMA**	**Atrophy grade at diagnosis (Marsh criteria)**
**Celiac patients**	aCD-patient	2	1	1	+	+	II-III
	GFD-patient	2	1	1	+	+	II-III
**Non-Celiac patients**	GP-patient	2	1	1	+/-	- (*)	M.M

Active celiac disease (aCD-patients), celiac disease on a gluten-free diet (GFD-patients), healthy controls (C-individuals) and other gastrointestinal pathology (GP-patients).and (B) plasma samples.

(*) Serological test were performed only in genetically susceptible patients.

M.M = Mild mucosal alterations non compatibles with CD.

####  Plasma samples

Plasma samples were obtained from blood samples treated with EDTA and were obtained according to the same inclusion criteria than tissue samples. Plasma samples (Table 2) were collected from 81 children [31 aCD-patients, 17 GFD-patients, 10 C-individuals and 23 GP-patients] and from 101 adults [16 aCD-patients, 12 GFD-patients, 27 C-individuals and 46 GP-patients], In all cases plasma samples were immediately stored at -20°C until tested. 

**Table 2:** Patients groups from plasma samples included in this study according to revised ESPGHAN criteria.

	**Study patients**	**n**	**Children**	**Adults**	**HLA DQ2/DQ8**	**IgA-tTG/EMA**	**Atrophy grade at diagnosis (Marsh criteria)**
**Celiac patients**	aCD-patient	47	31	16	+	+	II-III
	GFD-patient	29	17	12	+	+	II-III
**Non-Celiac patients**	C-individual	37	10	27	+/-	- (*)	N.D
	GP-patient	69	23	46	+/-	- (*)	N.D/M.M

Active celiac disease (aCD-patients), celiac disease on a gluten-free diet (GFD-patients), healthy controls (C-individuals) and other gastrointestinal pathology (GP-patients).

(*) Serological test were performed only in genetically susceptible patients.

N.D = Not determined.

M.M = Mild mucosal alterations non compatibles with CD.

### 2: HLA Genotyping

 Genomic DNA was purified from whole blood (UltraClean® Blood DNA Isolation Kit, Mo Bio, California, USA) and HLA-DQ2 genotyping performed by PCR amplification using sequence specific primers (MWG Biotech, Ebersberg, Germany) and the Kapa Taq kit (Kapa Biosystems, Boston, USA) [14]. Analysis of HLA-DQ8 haplotype was only performed in those DQ2 negative patients, using the DQB1 Only SSP2LQB1 kit (Micro SSP^TM^ DNA Typing Trays, One Lambda InC, California, USA).

### 3: IgA-tTG antibodies

 IgA-tTG Abs levels were analyzed in plasma samples by the available comercial kit (EliA CeliKey IgA, Phadia, Madrid, Spain) using an UniCAP 100^Є^ Instrument Version 1.0 (Pharmacia Diagnostics AB, Uppsala, Sweden).

### 4: Zymogram analysis

 Proteins from biopsy samples were isolated using the Trizol Reagent (Applied Biosystems, California, USA) according to the manufacturer’s protocol and quantified by the Bradford reaction. A total of 8 µg of protein samples were subsequently separated in 15% acrilamide/bisacrilamide (37.5:1) gel electrophoresis gliadin zymograms containing 0.1% of protein (gliadin, Sigma-Aldrich, Missouri, USA) using a mini-Protean II system (BioRad Laboratories Inc, California, USA). Gels were then washed twice (t=15’ each) with 2.5% Triton-X-100 to remove SDS and allow protein renaturalization, and incubated overnight at 37°C with the reaction buffer (50 mM Tris, 5 mM CaCl_2_, pH 7.5). Protease activity was revealed by staining with 0.1% Coomassie Brilliant Blue R-250 (Bio-Rad, California, USA) in a mixture of acetic acid:methanol:water (1:3:6), and destaining in the same solution mixture without the dye. A molecular weight marker was loaded in every gel (Kaleidoscope pre-stained standard, BioRad, California, USA). Additionally, stacking gel was kept during all the process since the lack of staining, compared to the running gel, was used as an internal control. All samples were separated at 200 Volts for 50 min in the running gel. 

### 5: Fingerprinting and ion-trap Mass Spectometry

 Duodenal CD specific proteases (26 and 82 kDa) from a previously described gliadin-degrading pattern [11] were removed from the substrate gel after electrophoresis and analyzed by a combined fingerprinting using a MALDI-TOF Reflex™ IV Bruker (Bruker-Franzen Analytic GmbH, Bremen, Germany) and ion-trap MS on a 3000^plus^ (Bruker Daltonics, Bremen, Germany) coupled to a Famos-Switchos-Ultimate (LCPackings, Amsterdam, The Netherlands) chromatographic system. 

### 6: Protein and peptide sequence analysis

Protein identification was carried out by searching in the database of the National Center for Biotechnology Information (NCBI) using BLASTP algorithm v.2.2.20. Merops peptidase database (v 9.8) was used to analyze the cleaving points of the identified peptides.

### 7: Synthesis of peptides

 Different peptides (native and deamidated) and terminal modifications (i.e. biotinylated peptides) derived from gliadin 8-mer sequence (Table 3) were supplied by Biomedal SL (Seville, Spain). 

**Table 3:** Peptides derived from gliadin 8-mer sequence supplied by Biomedal S.L for the development and standardization of the ELISA.

**Peptide**	**Sequence**
**PEP-BIO 1**	FPLQPQQPK-BIOTIN
**PEP-BIO 2**	FPLQPEQPK-BIOTIN
**PEP 1**	CGG FPLQPQQP GGG FPLQPQQP
**PEP 2**	CGG FPLQPEPQ GGG FPLQPEQP GGG FPLQPEQP
**PEP 3**	CGG FPLQPEQP GGG FPLQPEQP
**PEP 4**	CGG FPLQPEQP
**PEP 5**	CGG QPFPQPELPFP GGG FPLQPEQP
**PEP 6**	CGG PEQPYPQP GGG QPFPQPELPFP GGG FPLQPEQP
**PEP 7**	PALM-GGG FPLQPEQP
**PEP 8**	PALM-FPLQPEQP GGG FPLQPEQP
**PEP 9**	PALM-QPFPQPEQPFPQPELPFP GGG FPLQPEQP
**PEP 10**	Cyclic FPLQPEQP (head to tail)

Two biotynilated peptides (native and deamidated) and ten different combinations of sequence and terminal modifications.

PALM: Palmitic acid.

### 8: Enzyme-linked immunosorbent assay (ELISA)

 Maxisorp microtitre plates (Nunc, Roskilde, Denmark) were coated with a mix of 2 synthetic peptides (Biomedal SL, Spain) in phosphate buffer (pH 7.4) and incubated overnight at 4°C. The plates were washed with phosphate buffered saline (PBS)-Tween 20 and blocked with a solution (PBS-5% non-fat dry milk) for 1 h at room temperature (RT). Next, plasma samples (1/50) were added to the wells. After 1 h of incubation at RT, the plates were washed and horseradish peroxidase-conjugated rabbit anti-human IgA (catalog. No. P0216, DakoCytomation) was added (1/500) to duplicate wells and incubated for 1 h at RT. Then the plates were washed, and substrate solution (TMB, Sigma) was added. After 30 min of incubation at RT in the dark, the reaction was stopped with 0.3 M sulphuric acid, and the absorbance at 450 nm was measured (microplate reader UVM340, Asys Hitech GmbH, Eugendorf, Austria).

### 9: Statistical analysis

 Each plasma sample was analyzed by triplicate and the mean value calculated. Positive and negative controls (CD-patients and C-individuals, respectively) and buffer blanks were included in each assay. An absorbance cutoff value was defined for each age group. It was calculated using the GP-patients (mean ± 2 x standard deviation) with the aim of discriminate, with the higher specificity possible, between that group and CD-patients. Only samples with an absorbance value ≥ cutoff value were considered positives. Unpaired, two-tailed Student’s t-test was applied and *p*<0.05 was considered as statistically significant. Specificity [(true negatives / (true negatives + false positives)) x 100]s and sensitivity [true positives / (true positives + false negatives)] x 100] were calculated for each age group. 

## Results

### 1: Duodenal gliadin-degrading proteases specific from celiac patients release an 8-mer gliadin peptide

 Gliadin zymogram analysis confirmed that the gliadin-degrading protease pattern was specifically found in the duodenum of all 4 CD patients irrespectively of their status (aCD/GFD) or age (adults/children) while remained absent in protein samples obtained from non-CD pathogenic control (Figure S1) as previously reported [11]. In order to identify gliadin peptides specifically released by such enzymes, fingerprinting was applied to the most intense gliadin-degrading band obtained from the zymogram matrix (26 kDa) from a GFD-patient. The fingerprinting profiles did not allow any match with any known protein. Therefore, a secondary MALDI-TOF ion-trap MS was applied and 3 peptides (8-, 15- and 18-mer) were identified (Table 4 and Figure S2).

**Table 4:** Peptides identified by ion-trap mass spectrometry analysis of the 26 kDa degradation band: 8-mer, 15-mer and 18-mer; and their alignment with the original sequence from the proteins ω- and γ-gliadin and LMW glutenin from which they proceeded.

**Peptide**	**Prolamin**	**Sequence alignment**
**8-mer**		FPLQPQQP
	ω-gliadin	-**S**.**F**PLQPQQ**P**.**F**-
**15-mer**		PFIQPSLQQQLNPCK
	γ-gliadin	**-P**.**P**FIQPSLQQQLNPCK.N-
**18-mer**		VFLQQQCSPVAMPQSLAR
	LMW-glutenin	-K.VFLQQQCSPVAMPQSLAR.S-

In bold, cleaving points that could not had been generated through the technical process involving trypsin digestion and therefore likely to be derived from biological protease activities.

 To determinate the nature of the cleaving points generated in the 3 peptides resulting from the above analysis, we conducted a search on the peptidase database MEROPS [15]. As shown in Table 4, the C-end of the 15-mer peptide and both ends of the 18-mer peptide were potential cleaving points for trypsin so they may have been generated by the trypsin digestion applied during MALDI-TOF analysis. However, the N-end of the 15-mer peptide and both ends of the 8-mer peptide lack any described trypsin cleaving point so they did not seem to have been released during technical processing of the samples but on the contrary were likely to have been released by the action of the CD-specific duodenal gliadin-degrading proteases. A second independent analysis was also performed on the 82 kDa gliadin-degrading protease obtained from an aCD-patient revealing again the 8-mer gliadin peptide (Table 4). Since such peptide was found in two independent experiments and its cleaving points were likely to be derived from the CD-specific duodenal gliadin degrading proteases we decided to study it in depth.

### 2: Identification of the 8-mer peptide in proteins from toxic cereals for celiac patients

 The sequence alignment made with the BLASTP program of the NCBI server [16] revealed several homologous sequences to the 8-mer gliadin peptide which remained restricted to prolamin and glutenin proteins from cereals which are toxic to CD patients like wheat, rye and barley species. None of the 8-mer homologous sequences were found in non toxic cereals to CD patients, i.e. rice or maize. A full length copy of the 8-mer peptide was identified in 12 different species from the true grasses genera: *Hordeum, Triticum, Lophopyrum, Aegilops* and *Secale*. The 8-mer was more abundant in prolamins from wheat (ω-gliadin and ω-secalin in *Triticum*) with 196 peptides matched. Results are detailed in Table 5 and 6.

**Table 5:** Sequence alignment searches using the BLASTP algorithm v.2.2.20 in NCBI protein database with the 8-mer (FPLQPQQP) gliadin peptide.

**Prolamins**	**Closest relative protein**	**Accession**	**Score**	**Peptides matched**
**Hordein**	C-hordein storage protein, partial (*Hordeum vulgare*)	gi|123461	152	1
	B3-hordein (*Hordeum vulgare*)	gi|123459	137	1
	C-hordein (*Hordeum vulgare*)	gi|442524	582	2
	C-hordein (*Hordeum vulgare* subsp. *vulgare*)	gi|442524	659	3
**Glutenin**	D-type LMW* glutenin subunit (*Triticum aestivum*)	gi|208605344	724	3
**γ-gliadin**	γ-gliadin (*Trtiticum aestivum*)	gi|379319141	223	1
**ω-gliadin**	ω-gliadin storage protein (*Triticum aestivum*)	gi|10444084	489	3
	ω-gliadin partial (*Triticum aestivum*)	gi|224747073	647	3
	ω-gliadin partial (*Triticum aestivum*)	gi|224746065	621	5
	ω-gliadin partial (*Triticum aestivum*)	gi|224747071	716	5
	Putative ω-gliadin (*Triticum aestivum*)	gi|63252971	775	3
	ω-gliadin partial (*Triticum aestivum*)	gi|224747067	799	6
	ω-gliadin partial (*Triticum urartu*)	gi|294998471	483	4
	ω-gliadin partial (*Triticum urartu*)	gi|294998469	647	3
	ω-gliadin partial (*Triticum urartu*)	gi|294998467	685	5
	ω-gliadin partial (*Triticum urartu*)	gi|294998465	679	6
	ω-gliadin partial (*Triticum monococcum* subsp. *aegilopoides*)	gi|294998463	540	1
	ω-gliadin partial (*Triticum monococcum* subsp. *aegilopoides*)	gi|294998461	631	1
	ω-gliadin partial (*Triticum monococcum*)	gi|294998449	674	2
	ω-gliadin partial (*Triticum monococcum*)	gi|294998453	645	1
	ω-gliadin partial (*Triticum monococcum*)	gi|294998455	567	2
	ω-gliadin partial (*Triticum monococcum*)	gi|294998457	713	1
	ω-gliadin partial (*Triticum monococcum*)	gi|294998451	647	1
	ω-gliadin partial (*Triticum turgidum* subsp. *paleocolchicum*)	gi|281398157	678	2
	ω-gliadin partial (*Lophopyrum elongatum*)	gi|224747083	647	1
	ω-gliadin partial (*Lophopyrum elongatum*)	gi|224747089	666	1
	ω-gliadin partial (*Lophopyrum elongatum*)	gi|224747087	656	2
	ω-gliadin partial (*Triticum aestivum x Lophopyrum elongatum*)	gi|224747080	647	1
	ω-gliadin partial (*Triticum aestivum x Lophopyrum elongatum*)	gi|224747075	652	4
	ω-gliadin partial (*Triticum aestivum x Lophopyrum elongatum*)	gi|224747078	703	6
	ω-gliadin (*Aegilops markgrafii*)	gi|410025837	612	2
	ω-gliadin (*Aegilops markgrafii*)	gi|410025835	578	2
	ω-gliadin (*Aegilops markgrafii*)	gi|410025829	668	3
	ω-gliadin (*Aegilops markgrafii*)	gi|410025833	676	3
	ω-gliadin (*Aegilops markgrafii*)	gi|410025831	759	4
	ω-gliadin partial (*Aegilops tauschii*)	gi|373430784	592	1
	ω-gliadin partial (*Aegilops tauschii*)	gi|50313199	781	3

Protein sequences are grouped according to the type of prolamin (hordein, glutenin, γ-gliadin and ω-gliadin) and the number of 8-mer peptide matched in each sequence is shown. LMW (Low Molecular Weight).

**Table 6:** Sequence alignment searches using the BLASTP algorithm v.2.2.20 in NCBI protein database with the 8-mer (FPLQPQQP) gliadin peptide.

**Prolamins**	**Closest relative protein**	**Accession**	**Score**	**Peptides matched**
**ω-secalin**	Putative ω-secalin (*Triticum aestivum*)	gi|226247119	196	1
	Putative ω-secalin (*Triticum aestivum*)	gi|226247117	202	1
	Putative ω-secalin (*Triticum aestivum*)	gi|226247115	286	2
	Putative ω-secalin (*Triticum aestivum*)	gi|226247113	205	2
	Putative ω-secalin (*Triticum aestivum*)	gi|226247111	290	1
	Putative ω-secalin (*Triticum aestivum*)	gi|226247109	315	2
	Putative ω-secalin (*Triticum aestivum*)	gi|226247107	328	1
	Putative ω-secalin (*Triticum aestivum*)	gi|226247105	313	1
	Putative ω-secalin (*Triticum aestivum*)	gi|226247103	327	1
	Putative ω-secalin (*Triticum aestivum*)	gi|226247101	310	1
	Putative ω-secalin (*Triticum aestivum*)	gi|226247099	359	2
	Putative ω-secalin (*Triticum aestivum*)	gi|226247097	289	3
	Putative ω-secalin (*Triticum aestivum*)	gi|226247095	317	4
	Putative ω-secalin (*Triticum aestivum*)	gi|226247093	392	3
	Putative ω-secalin (*Triticum aestivum*)	gi|226247091	334	2
	Putative ω-secalin (*Triticum aestivum*)	gi|225625618	295	2
	Putative ω-secalin (*Triticum aestivum*)	gi|225625624	366	3
	Putative ω-secalin (*Triticum aestivum*)	gi|225625620	399	2
	Putative ω-secalin (*Triticum aestivum*)	gi|225625622	416	3
	Putative ω-secalin (*Triticum aestivum*)	gi|225625614	463	1
	Putative ω-secalin (*Triticum aestivum*)	gi|225625616	444	4
	ω-secalin (*Triticum aestivum*)	gi|229610236	516	2
	ω-secalin (*Triticum aestivum*)	gi|229610230	573	4
	ω-secalin (*Triticum aestivum*)	gi|229610226	700	5
	ω-secalin (*Triticum aestivum*)	gi|229610238	698	4
	ω-secalin (*Triticum aestivum*)	gi|229610234	699	5
	ω-secalin (*Triticum aestivum*)	gi|229610232	577	5
	ω-secalin (*Triticum aestivum*)	gi|229610228	703	6
	Putative ω-secalin (*Triticum aestivum*)	gi|225625626	708	6
	ω-secalin (*Secale cereale*)	gi|2145025	692	4
	Sec1 precursor (*Secale cereale*)	gi|21202	677	6
	ω-secalin (*Secale cereale*)	gi|229610198	661	4
	ω-secalin (*Secale cereale*)	gi|229610196	687	6
	ω-secalin (*Secale cereale*)	gi|229610194	654	5
	ω-secalin (*Secale cereale*)	gi|229610192	685	5
	ω-secalin (*Secale cereale*)	gi|229610190	664	5
	Sec1 precursor (*Secale cereale*)	gi|21204	669	6
	ω-secalin (*Triticum aestivum x Secale cereale*)	gi|229610219	661	6
	ω-secalin (*Triticum aestivum x Secale cereale*)	gi|229610217	700	6
	ω-secalin (*Triticum aestivum x Secale cereale*)	gi|229610215	645	4
	ω-secalin (*Secale cereale x Triticum durum*)	gi|229610210	704	6
	ω-secalin (*Secale cereale x Triticum durum*)	gi|229610208	696	6
	ω-secalin (*Secale cereale x Triticum durum*)	gi|229610206	687	6
	ω-secalin (*Secale cereale x Triticum durum*)	gi|229610204	699	6

Protein sequences from ω-secalin and the number of 8-mer peptide matched in each sequence is shown.

### 3: The 8-mer peptide overlaps with 3 known gluten T cell epitopes

 We carried out a search for the known CD relevant gluten T cell epitopes restricted by HLA-DQ molecules [6] in proteins containing the 8-mer peptide. The 8-mer peptide appeared overlapped or next to three T-cell epitopes (Table 7), and the most repeated was QQPFPQQPQ [17] which overlapped in 34.5% of the 8-mer peptides matched in ω-gliadin, 13.1% in ω-secalin, 14.3% in hordein and 33.4% in glutenin sequences. Two more T-cell epitopes were close to the 8-mer peptide despite not overlapping, QQPQQPFPQ and PFPQPQQPF [17,18], which were more repeated in ω-secalin.

**Table 7:** Identification and analysis of the alignment between the 8-mer peptide and three gluten T-cell epitopes identified previously.

**Prolamins**	**T-cell epitopes**	**Peptides**
**γ-gliadin**	QQPQQPFPQ	QQP.**FPLQPQQP**.FPQPQQPQQPFPQ.X
**ω-gliadin**	QQPFPQQPQ	QQX.**FPLQPQQP**.FPQQPQQP
	QQPQQPFPQ	QQX.**FPLQPQQP**.FPQQPQQPFPQ.X
	QQPFPQQPQ	QLQQPFPQQPQQP.**FPLQPQQP**.FP
**ω-secalin**	QQPFPQQPQ	QQX.**FPLQPQQP**.FPQQPQ
	PFPQPQQPF	QQX.**FPLQPQQP**.SPQQPQLPFPQPQQPFVVVV
**Hordein**	QQPFPQQPQ	QQP.**FPLQPQQP**.FPQQPQQPFPQPQQPFR
	PFPQPQQPF	QQPFPQPQQP.**FPLQPQQP**.FP
**Glutenin**	QQPFPQQPQ	S.**FPLQPQQP**.FPQQPQQPFPQP
	QQPQQPFPQ	QQPQQPFPQQPQQP.**FPLQPQQP**.FP

In bold, 8-mer peptide sequence and T-cell epitopes are indicated by underlining. BLASTP (v 2.2.20) and Merops (v 9.8) databases were used.

### 4: Development and standardization of ELISA based on the 8-mer peptide

 HLA-DQ2 and DQ8 molecules have preference for binding of negatively charged amino acid residues in certain binding pockets [19]. In vivo, enzyme tTG mediates deamidation of some glutamine residues into glutamic acid acquiring a negative charge and rendering peptides more suitable for presentation by DQ2&DQ8 molecules [7]. Thus, we studied the presence in plasma samples from CD patients of IgA Abs against both the native and deamidated 8-mer peptides. It has been shown that the targeting of Q residues in peptides is strongly influenced by the position of C-terminally located P residues. Whereas a Q residue in the QXP consensus sequence is targeted by tTG, Q residues in a QP or QXXP sequence motif are not [20,21]. Therefore, we studied the effect on the reactivity of the 8-mer peptide after its deamidation in position 6 (FPLQPEQP). 

 Two synthetic biotynilated peptides (native and deamidated, Table 3) were used in streptavidin coated plates to study the presence of IgA antibodies in plasma samples from CD patients (Biomedal S.L). The levels of IgA Abs against deamidated 8-mer peptide in plasma samples from celiac patients was three fold higher than the one observed in the same patients with the native 8-mer peptide (results not shown). The antigen-antibody complex seemed to be stronger when the deamidated 8-mer peptide was used than with the native sequence. In order to improve the detection of antibodies by ELISA, ten different combinations of sequence and terminal modifications were designed (Table 3) and containing, all but one, at least one copy of deamidated gliadin 8-mer epitopes (Biomedal S.L). Finally, the combination of two peptides was selected as the optimum recombinant antigen to coat the microtiter plates for the standardization of the ELISA.

### 5: Detection of IgA anti-DGP 8-mer antibodies in plasma samples from celiac children

**Figure 1:**
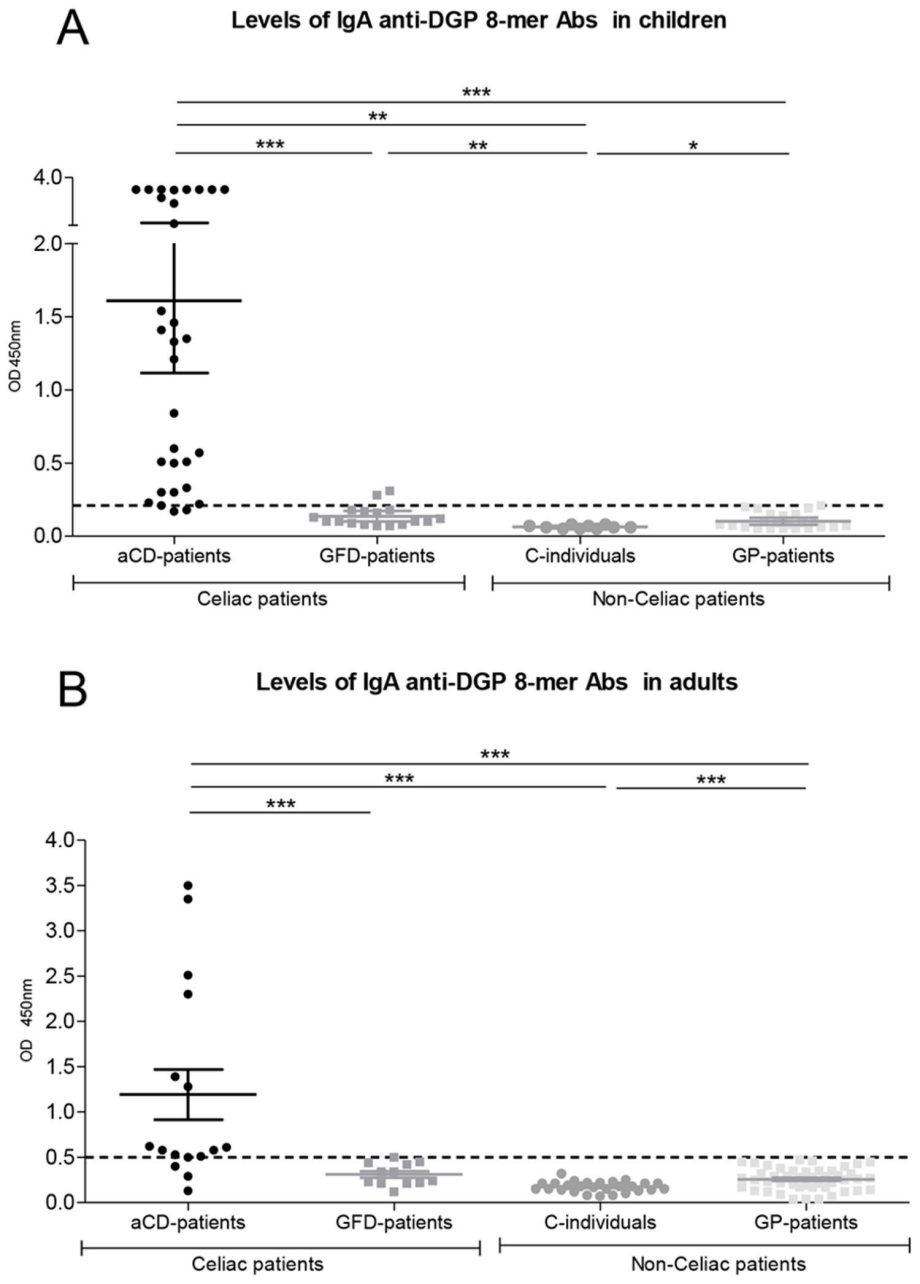
Detection of IgA anti-DGP 8-mer antibodies in plasma samples from children (A) and adults (B). Study groups: active celiac disease (aCD-patients), on a gluten-free diet (GFD-patients), healthy controls (C-individuals) and other gastrointestinal pathology (GP-patients). Each point represents the mean absorbance value of one patient at O.D 450 nm. According to the cutoff value (0.21 in children and 0.5 in adults, pointed line), patients with a higher absorbance value were considered positive for IgA anti-DGP 8-mer Abs, while those with lower absorbance values were considered negative. * p<0.05, ** p<0.01 and *** p<0.001 (Unpaired, two-tailed Student’s t-test). A- Among pediatric population 90.6% of aCD, 10.5% of GFD and 4.3% of GP-patients were positive for IgA anti-DGP 8-mer antibodies, and 9.4% of aCD-patients, 89.5% of GFD-patients, 100% of C-individuals and 95.7% of GP-patients were negative. **B**- In adult population, 18.7% of aCD, 100% of GFD-patients, C-individuals and GP-patients were positive for IgA anti-DGP 8-mer, while 18.7% of aCD and 100% of GFD-, C- and GP-patients were negative.

 The ELISA test designed in this article allowed us to identify IgA Abs which recognize DGP 8-mer as a specific antigen in the plasma from CD patients. Having optimized the technique, we first studied the presence of specific IgA anti-DGP 8-mer Abs in plasma from celiac children (Table 2, Figure 1 (A)). The absorbance values (mean ± SEM) at O.D 450 nm obtained for each group of patients were: aCD-patients (n=31) 1.61 ± 0.24, GFD-patients (n=17) 0.14 ± 0.02, C-individuals (n=10) 0.06 ± 0.003 and GP-patients (n=23) 0.10 ± 0.01. According to the cutoff value calculated for the pediatric population (0.21), among aCD-patients (n=31), there were 29 positives and 2 negatives for IgA anti-DGP 8-mer Abs. In the case of GFD-patients, only 2 children were positives for IgA-8-mer Abs. Very low levels of IgA anti-DGP 8-mer Abs were detected in 1 out of 23 GP-patients. The remaining 22 GP-patients and all C-individuals had negative levels for IgA anti-DGP 8-mer Abs. 

 Comparison of the mean absorbance levels between aCD-patients and the remainder groups showed significant differences (p<0.001). IgA anti-DGP 8-mer Abs not only differentiated between CD patients and non-CD patients, but also between active celiac patients and those celiac patients who have initiated a GFD. The modified ELISA method used for detect IgA anti-DGP 8-mer antibodies showed 94% of specificity and 93.5% of sensitivity in children. 

### 6: Detection of IgA anti-DGP 8-mer antibodies in plasma samples from celiac adults

 CD is a common disorder in children and adults, and shows age-related differences at initial diagnosis. Given that IgA anti-TGt levels correlate both with the degree of villous atrophy and with the patient’s age [22], we studied if the IgA anti-DGP 8-mer ELISA test could be also applied to adult patients (Table 2).

 Results obtained in the adult population are shown in Figure 1 (B). The absorbance values at O.D 450 nm obtained for each group were (mean ± SEM): aCD-patients (n=16) 1.19 ± 0.28, GFD-patients (n=12) 0.31± 0.03, C-individuals (n=27) 0.17 ± 0.01; and GP-patients (n=46) 0.26 ± 0.01. According to the cutoff value calculated for the adult population (0.5), among the group of aCD- patients (n=16), there were 13 positives and 3 negatives for IgA anti-DGP 8-mer Abs. All GFD-patients (n=12) had negative levels of antibodies, except one. IgA anti-DGP 8-mer Abs were not detected in any of the non-celiac patients. 

 When mean absorbance levels obtained by aCD-patients and the remainder groups were compared, significant differences were obtained (p<0.001). The ELISA test showed a specificity of 98.8% and a sensitivity of 81.3% in adult population.

## Discussion

 We have previously described a CD-specific duodenal gliadin-degrading pattern with seven proteases with molecular weight’s ranging from 20 kDa to 92 kDa [11]. In this work we describe for the first time to our knowledge a new gliadin-derived 8-mer peptide (FPLQPQQP) released after gliadin digestion by duodenal CD-specific proteases. In addition, it remained restricted to prolamin and glutenin proteins from toxic cereals to CD patients (wheat, barley and rye). Among the more than 250 homologous sequences to the 8-mer peptide matched most of them was expressed in ω-prolamins (gliadin and secalin). To study if the 8-mer peptide could be found in already described toxic peptides for CD patients, we carried out a search for the known CD relevant gluten T-cell epitopes restricted by HLA-DQ molecules [6]. Since several HLA-DQ restricted gluten T-cell epitopes overlapped or were close to the 8-mer peptide, we wondered if IgA antibodies against the 8-mer peptide could be found in the plasma from CD patients. 

 The first ELISA assays in which native and deamidated 8-mer peptide were compared, showed an increase in the IgA anti 8-mer levels in CD patients when the peptide was deamidated in position 6. The increase in the reactivity of gliadin peptides in CD has been observed in several studies of native and deamidated peptides [23,24], and the effect of deamidation has been confirmed in the induction of a Th2 response in a mice model [25]. For that reason, we designed different peptides containing at least one copy of the DGP 8-mer and combined with terminal modifications (Biomedal S.L) in order to select those more effective in the cover of the plate and in the binding to the antibodies. Finally, the combination of two peptides was selected as the optimum recombinant antigen to develop the assay which allowed as to detect IgA anti-DGP 8-mer in CD-patients.

 Among pediatric population, levels of IgA anti-DGP 8-mer Abs were negative only in 2 aCD-patients (aCD-30 and aCD-31 in Table 8). A possible explanation for one of these cases is that the patient number 31 has a celiac sister so it is possible that he was already following (at least partially) either a GFD or a low-gluten containing diet as most of close relatives of CD patients do. Regarding GFD-patients, IgA anti-DGP 8-mer Abs were positive only in 2 of 17 children. One of them (GFD-32 in Table 9) turned out to be a case of not response to the gluten-free diet in addition to suffering prolonged diarrhea and also having negative IgA anti-tTG levels. The other child on a GFD with positive IgA anti-DGP 8-mer levels (GFD-33 in Table 9), had IgA anti-tTG levels one unit above the cutoff value, and 6 months later, IgA anti-tTG plasma levels became negative.

**Table 8:** Clinical data from active celiac patients (n=31) from the pediatric population (n=48).

**Sample**	**Sex**	**Age**	**HLA DQ2/DQ8**	**IgA anti-tTG**	**IgA anti-DGP 8-mer**	**Atrophy grade at diagnosis (Marsh criteria)**
**aCD-1**	M	2	+	+	+	III
**aCD-2**	F	2	+	+	+	III
**aCD-3**	F	3	+	+	+	III
**aCD-4**	F	1	+	+	+	III
**aCD-5**	M	1	+	+	+	II
**aCD-6**	F	3	+	+	+	III
**aCD-7**	M	1	+	+	+	N.D
**aCD-8**	F	2	+	+	+	N.D
**aCD-9**	F	3	+	+	+	III
**aCD-10**	M	2	+	+	+	III
**aCD-11**	M	2	+	+	+	III
**aCD-12**	F	2	+	+	+	M.M
**aCD-13**	F	3	+	+	+	III
**aCD-14**	F	6	+	+	+	III
**aCD-15**	F	1	+	+	+	N.D
**aCD-16**	F	2	+	+	+	III
**aCD-17**	F	9	+	+	+	III
**aCD-18**	F	2	+	+	+	III
**aCD-19**	M	8	+	+	+	N.D
**aCD-20**	F	2	+	+	+	N.D
**aCD-21**	M	3	+	+	+	III
**aCD-22**	F	2	+	+	+	III
**aCD-23**	F	3	+	+	+	III
**aCD-24**	F	3	+	+	+	III
**aCD-25**	F	2	+	+	+	III
**aCD-26**	F	10	+	+	+	III
**aCD-27**	F	10	+	+	+	I
**aCD-28**	F	1	+	+	+	III
**aCD-29**	M	11	+	+	+	III
**aCD-30**	M	1	+	+	-	III
**aCD-31**	F	9	+	+	-	III

Table shows the following information: sex (M: male and F: female), age (from 1 to 12 years), HLA DQ2/DQ8, IgA anti-tTG, IgA anti-DGP 8-mer and Marsh scale of the severity of lesion at diagnosis (from Marsh I to Marsh III). N.D (Not determined) and M.M (mild mucosal alterations).

**Table 9:** Clinical data from celiac patients on a gluten-free diet (n=17) from the pediatric population (n=48).

**Sample**	**Sex**	**Age**	**HLA DQ2/DQ8**	**IgA anti-tTG**	**IgA anti-DGP 8-mer**	**Atrophy grade at diagnosis (Marsh criteria)**	**Months on GFD**
**GFD-32**	F	10	+	-	+	III	N.R
**GFD-33**	F	7	+	+	+	III	4
**GFD-34**	F	6	+	-	-	III	4
**GFD-35**	F	2	+	+	-	N.D	1.5
**GFD-36**	M	4	+	+	-	III	3
**GFD-37**	F	2	+	-	-	N.D	6
**GFD-38**	F	3	+	+	-	III	3
**GFD-39**	M	4	+	+	-	III	4
**GFD-40**	F	2	+	-	-	N.D	10
**GFD-41**	F	2	+	-	-	N.D	6
**GFD-42**	M	5	+	+	-	III	3
**GFD-43**	M	2	+	-	-	III	2
**GFD-44**	F	3	+	+	-	II	3
**GFD-45**	M	1	+	+	-	III	12
**GFD-46**	M	12	+	+	-	N.D	5
**GFD-47**	F	11	+	+	-	N.D	7
**GFD-48**	M	2	+	+	-	N.D	1

Table shows the following information: sex (M: male and F: female), age (from 1 to 12 years), HLA DQ2/DQ8, IgA anti-tTG, IgA anti-DGP 8-mer and Marsh scale of the severity of lesion at diagnosis (from Marsh I to Marsh III). N.D (Not determined) and M.M (mild mucosal alterations). The time on GFD is shown in months, except one patient who did not response to the GFD (N.R).

 Ten children had positive levels of IgA anti-tTG despite being on a GFD and having clinical recovery. However, all of them were negative for IgA anti-DGP 8-mer Abs suggesting the possible application of IgA anti-DGP 8-mer antibodies as a marker of GFD compliance in children. Nevertheless, further studies are required to confirm this point, including an assessment of the patients and a direct and controlled comparison of the tTG enzyme and DGP 8-mer peptide IgA antibodies, with direct controls of GFD compliance by fecal detection of gluten peptides [26].

 Although IgA anti-tTG antibodies have been proposed as markers of GFD compliance, their effectiveness is still not clear since they have been reported both well [27] or poorly [28] correlated with dietary transgressions. The normalization of IgA anti-tTG levels occurs, approximately, after 6 months on a strict GFD. Nevertheless, IgA anti-DGP 8-mer Abs were not detected in a child after a month on a GFD, while he was positive for IgA ant-tTG. In agreement with our data, Comino et al [26] estimated the time of total gluten toxic-peptide excretion to be between 2 and 6 days and the biological half-live for IgA antibodies in human plasma has been calculated in 5.9 and 4.5 days for IgA1 and IgA2 subclasses respectively [29]. 

 Specificity is the critical parameter to discriminate CD from other gastrointestinal pathologies, and sensitivity is crucial for finding new patients. Among the 23 GP-patients studied, only one patient, who was son of a CD woman, was positive for IgA anti-DGP 8-mer test. According to a recent report published in 2012 by the ESPGHAN [13] and other studies of antibodies against DGP [30,31], the specificity is ranged between 86.3% and 93.1%, while sensitivity is in the range of 80.7% to 95.1%. Since our results in pediatric population were similar to those observed in other DGP studies, we next wondered if our ELISA test could also be applied to the adult population where CD is more frequently diagnosed in older population (20% of the celiac adults are older than 65 years). Therefore, classical gastrointestinal symptoms characteristics of CD are usually accompanied by other manifestations and pathologies usually linked with the age including liver cirrhosis, inflammatory bowel disease and diabetes mellitus, which have been related with CD in several studies [32–36]. Since CD diagnosis in adults is getting more complicated and false positive reactions for IgA anti-tTG have been reported we also studied 46 plasma samples from adult non-celiac patients but with other gastrointestinal pathology. All of them were negative for IgA anti-DGP 8-mer while some of them had positive levels of IgA anti-tTG revealing therefore a specificity of 100% in such cohort.

 The highest absorbance values in pediatric population (Figure 1 (A)) were almost 4 fold over those observed in the adult one (Figure 1 (B)). However IgA anti-DGP 8-mer antibodies could provide an additional degree of specificity in both populations, compared to other serological markers, due to the following reasons: tissue transglutaminase enzyme is expressed in a constitutive manner in everyone [37] because of its functions in apoptosis and cellular differentiation and anti-tTG antibodies may persist independently of the GFD compliance or could be generated in other pathologies. In addition, DGP usually are derived from whole gluten digested with commercial enzymes like pepsin, trypsin, chymotrypsin, elastase and carboxypeptidase and subsequently modified with TG2 [20]. On the other hand, the 8-mer peptide is generated by specific duodenal proteases of celiac patients.

## Conclusion

 In this study we have identified, for the first time to our knowledge plasma CD-specific IgA Abs that recognize as specific antigen a novel deamidated gliadin peptide (FPLQPEQP), derived from an 8-mer peptide generated during duodenal degradation of gliadin by CD-specific duodenal proteases. The native peptide (FPLQPQQP) remained restricted to prolamin and glutenin proteins from toxic cereals to CD patients and was particularly expressed in ω-prolamins. The 8-mer peptide overlapped with a previously identified gluten T cell epitope and its ability to develop a humoral immune response in vivo in CD patients was confirmed by the detection of IgA anti-DGP 8-mer Abs in plasma samples from those patients.

 The DGP 8-mer could be used as a novel specific CD antigen for the diagnosis of CD and monitoring of the compliance of GFD. We have designed an ELISA test which detects IgA anti-DGP 8-mer Abs in plasma samples from CD-patients with a high specificity (94% in children and 98.8% in adults) and sensitivity (93.5% in children and 81.3% in adults). Future studies with larger number of samples and additional relevant data (i.e. use of novel tools for GFD compliance) will assess the full potential of the novel diagnostic tool for celiac diagnosis and management.

## Supporting Information

Highlights S1 **Significance of this study.** Significance of this study in the context of what is already known about this subject and the new findings described in this manuscript.
(DOC)

Figure S1 **the gliadin-degrading protease pattern.** Gliadin zymogram analysis (A) of whole protein duodenal biopsy explants from active celiac patients (aCD, lines 1 and 2), celiac patients on a gluten-free diet (GFD, lines 3 and 4) and patients with other gastrointestinal pathology but non-celiac disease (GP, lines 5 and 6). The gliadin-degrading protease pattern, characterized by 7 CD-specific proteases (from 92 to 20 kDa), was found in aCD and GFD patients while was absent in GP-patients. The fingerprinting and ion-trap mass spectrometry analyses of 26 kDa protease from a GFD-patient (line 3) and 82 kDa protease from an aCD-patient (line 2) allowed the identification of three peptides: 8-, 15- and 18-mer. (B) Sequences, cleaving points and alignments of the peptides identified and its corresponding prolamin. (C) Mass spectrum of the 8-mer peptide.
(DOC)

Figure S2 **the ion-trap mass spectrometry analysis.** Mass spectrum and sequences of 15- and 18-mer peptides identified by ion-trap mass spectrometry analysis of the 26 kDa protease obtained by gliadin zymogram analysis of the whole protein from a GFD-patient biopsy sample.
(DOC)
