# Supplementary material for: Detection of Specific IgA Antibodies against a Novel Deamidated 8-Mer Gliadin Peptide in Blood Plasma Samples from Celiac Patients
Source: PLoS One. 2013 Nov 22;8(11):e80982. doi: 10.1371/journal.pone.0080982 (PMC3838339; doi:10.1371/journal.pone.0080982)
Supplement: Highlights S1 — Significance of this study. Significance of this study in the context of what is already known about this subject and the new findings described in this manuscript. (DOC) [file pone.0080982.s001.doc]

# STUDY HIGHLIGHTS

# What is current knowledge

- Celiac disease (CD) is an enteropathy that is triggered by an environmental stimulus (gluten) in individuals with a genetic predisposition.
- CD diagnosis is based on an histological evaluation by duodenal biopsy, genetic susceptibility and the presence of serological markers. The identification of several gluten peptides that stimulate T cells from CD patients has allowed the develop of serological test to detect CD-specific antibodies against deamidated gliadin peptides (DGP).
- We have previously identified a CD-specific pattern of gliadin-degrading duodenal proteases that was absent in the duodenal mucosa of non celiac patients.

# What is new here

# We have identified a novel gliadin-derived 8-mer peptide that is restricted to prolamin and glutenin proteins from toxic cereals to CD patients and which is generated during gliadin degradation by CD-specific duodenal proteases.

# We have identified plasma CD-specific IgA antibodies that recognize the deamidated gliadin-derived 8-mer peptide as specific antigen.

- The IgA anti-DGP 8-mer ELISA test designed and developed in this study could be used as a novel tool in the diagnosis of CD and the control of GFD compliance.
