# Supplementary figures and images for: Detection of Specific IgA Antibodies against a Novel Deamidated 8-Mer Gliadin Peptide in Blood Plasma Samples from Celiac Patients
Source: PLoS One. 2013 Nov 22;8(11):e80982. doi: 10.1371/journal.pone.0080982 (PMC3838339; doi:10.1371/journal.pone.0080982)

**Figure S1:**

**
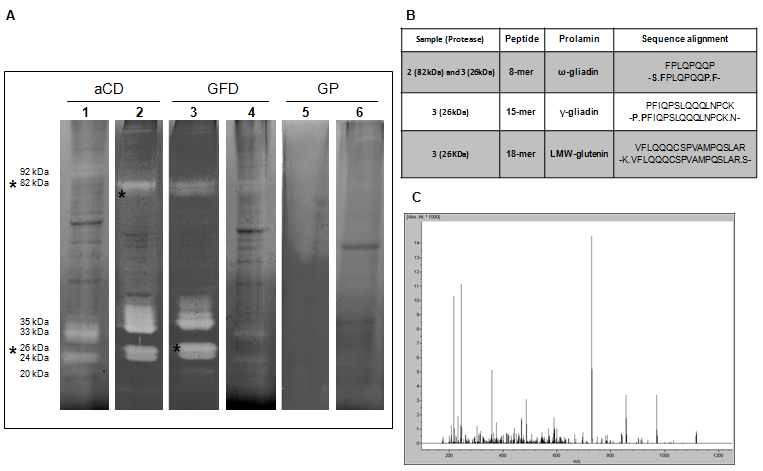
**

Supplement: Figure S1 — the gliadin-degrading protease pattern. Gliadin zymogram analysis (A) of whole protein duodenal biopsy explants from active celiac patients (aCD, lines 1 and 2), celiac patients on a gluten-free diet (GFD, lines 3 and 4) and patients with other gastrointestinal pathology but non-celiac disease (GP, lines 5 and 6). The gliadin-degrading protease pattern, characterized by 7 CD-specific proteases (from 92 to 20 kDa), was found in aCD and GFD patients while was absent in GP-patients. The fingerprinting and ion-trap mass spectrometry analyses of 26 kDa protease from a GFD-patient (line 3) and 82 kDa protease from an aCD-patient (line 2) allowed the identification of three peptides: 8-, 15- and 18-mer. (B) Sequences, cleaving points and alignments of the peptides identified and its corresponding prolamin. (C) Mass spectrum of the 8-mer peptide. (DOC) [file pone.0080982.s002.doc]
