# Supplementary material for: Detection of Specific IgA Antibodies against a Novel Deamidated 8-Mer Gliadin Peptide in Blood Plasma Samples from Celiac Patients
Source: PLoS One. 2013 Nov 22;8(11):e80982. doi: 10.1371/journal.pone.0080982 (PMC3838339; doi:10.1371/journal.pone.0080982)
Supplement: Figure S2 — the ion-trap mass spectrometry analysis. Mass spectrum and sequences of 15- and 18-mer peptides identified by ion-trap mass spectrometry analysis of the 26 kDa protease obtained by gliadin zymogram analysis of the whole protein from a GFD-patient biopsy sample. (DOC) [file pone.0080982.s003.doc]

**Figure S2**

**15-mer peptide 18-mer peptide**

PFIQPSLQQQLNPCK VFLQQQCSPVAMPQSLAR
